# Adult Thalamic Diffuse Midline Glioma, H3 K27-Altered, With *KRAS* Comutation and Potential Early Subependymal Dissemination

**DOI:** 10.14740/jmc5399

**Published:** 2026-10-02

**Authors:** Muralidhar Idamakanti, Rani Indrani Bijjam, Yasvin Onkarappa Mangala

**Affiliations:** a Adult Inpatient Medical Services (AIMS), Presbyterian Healthcare Services (PHS), Albuquerque, NM 87106, USA; b Department of Hematology/Oncology, Presbyterian Healthcare Services (PHS), Albuquerque, NM 87106, USA

**Keywords:** Adult diffuse midline glioma, *H3F3A* K27M and *KRAS* G12V comutation, MAPK pathway, Radiotherapy, Dordaviprone, Early CSF dissemination

## Abstract

Diffuse midline glioma (DMG) is a highly aggressive central nervous system neoplasm that predominantly affects pediatric populations and is associated with a dismal prognosis. Adult DMGs remain relatively uncommon and continue to pose significant diagnostic and therapeutic challenges. We present the case of a 54-year-old adult patient with a left thalamic DMG, H3 K27-altered, World Health Organization (WHO) grade 4, harboring pathogenic *H3F3A* K27M, *KRAS* G12V, and *PHF6* mutations. The patient presented with headaches, fatigue, imbalance, dizziness, and slowed dexterity. Imaging demonstrated a heterogeneously enhancing left thalamic lesion extending into the midbrain, with associated edema and third-ventricular compression. Stereotactic biopsy confirmed a DMG, H3 K27-altered. Given the surgically inaccessible location and the high risk of neurologic morbidity, the patient underwent definitive radiotherapy without concurrent temozolomide. Follow-up imaging showed an interval reduction in tumor size, consistent with treatment response; however, a new subependymal ventricular nodule raised concern for possible early dissemination. This case highlights the importance of molecular characterization in adult DMG and discusses the potential biological implications of a *KRAS* comutation in this rare entity. We also review the evolving literature on adult thalamic DMG, molecular alterations, therapeutic considerations, and emerging targeted therapies, including dordaviprone (ONC201).

## Introduction

Adult diffuse midline gliomas (DMGs) are rare World Health Organization (WHO) grade 4 gliomas, with an estimated annual incidence of about 2.3 cases per million adults, and account for only 5% of all adult diffuse gliomas [1–4]. DMGs are classically associated with pediatric diffuse intrinsic pontine gliomas (DIPGs), and adult cases are usually rare [3, 4]. Adult DMGs show significant clinicopathologic heterogeneity compared with pediatric disease, including differences in anatomical distribution, imaging characteristics, molecular alterations, and survival outcomes [3–5]. They can occur throughout adulthood, with most adult patients presenting between 18 and 40 years of age and a reported median age of approximately 36–38 years. There appears to be a slight male predominance [4, 5].

Adult DMGs arise within midline structures of the central nervous system, most frequently involving the thalamus, brainstem, and spinal cord. H3 K27 alterations are identified in approximately 15–60% of adult DMGs, a considerably lower frequency than the 80–90% reported in pediatric DMGs [4–6]. Emerging evidence suggests that the biological behavior of adult H3 K27-altered DMGs may differ from that of pediatric disease, with some studies reporting longer survival among adults harboring H3 K27M-mutant tumors compared with pediatric cases [6, 7]. Molecular characterization of adult DMG has revealed recurrent co-occurring genomic alterations involving *TP53*, *ATRX*, *EGFR*, *ACVR1*, and *PDGFRA* [7–9]. However, *KRAS* and *PHF6* mutations remain infrequently reported in DMG, and their clinical significance remains poorly understood. Emerging evidence suggests that MAPK pathway dysregulation may contribute to gliomagenesis and tumor progression in select glioma subsets [9].

We report a case of adult thalamic DMG, H3 K27-altered, harboring comutations in *KRAS* G12V and *PHF6* and variations in *PDFGRA*, with radiographic heterogeneity and possible early subependymal dissemination, and discuss the molecular, radiologic, and therapeutic implications in the context of current literature.

## Case Report

We report a case of a 54-year-old man with a medical history of gastroesophageal reflux disease (GERD), hepatic steatosis, mild intermittent asthma, nephrolithiasis, lumbar spondylosis, and a former tobacco use disorder who presented to the emergency room with fatigue, intermittent headaches, dizziness, imbalance, and slowed dexterity. Symptoms progressively worsened over 6–8 weeks, prompting admission, neurology evaluation, and neuroimaging.

Computed tomography (CT) of the head on presentation demonstrated a left thalamic/basal ganglia mass with surrounding edema and mass effect. Subsequent magnetic resonance imaging (MRI) of the brain revealed a heterogeneous intra-axial lesion centered within the left thalamus and extending into the left midbrain, measuring approximately 3.2 × 2.6 cm. The lesion demonstrated predominantly T1-hypointense signal (Fig. 1) with mixed T2 hypo- and hyperintense signal characteristics (Fig. 2). Significant surrounding vasogenic edema was noted. Considerable mass effect on the third ventricle was present, with compression and rightward deviation (Figs. 1–3).

**Figure 1:**
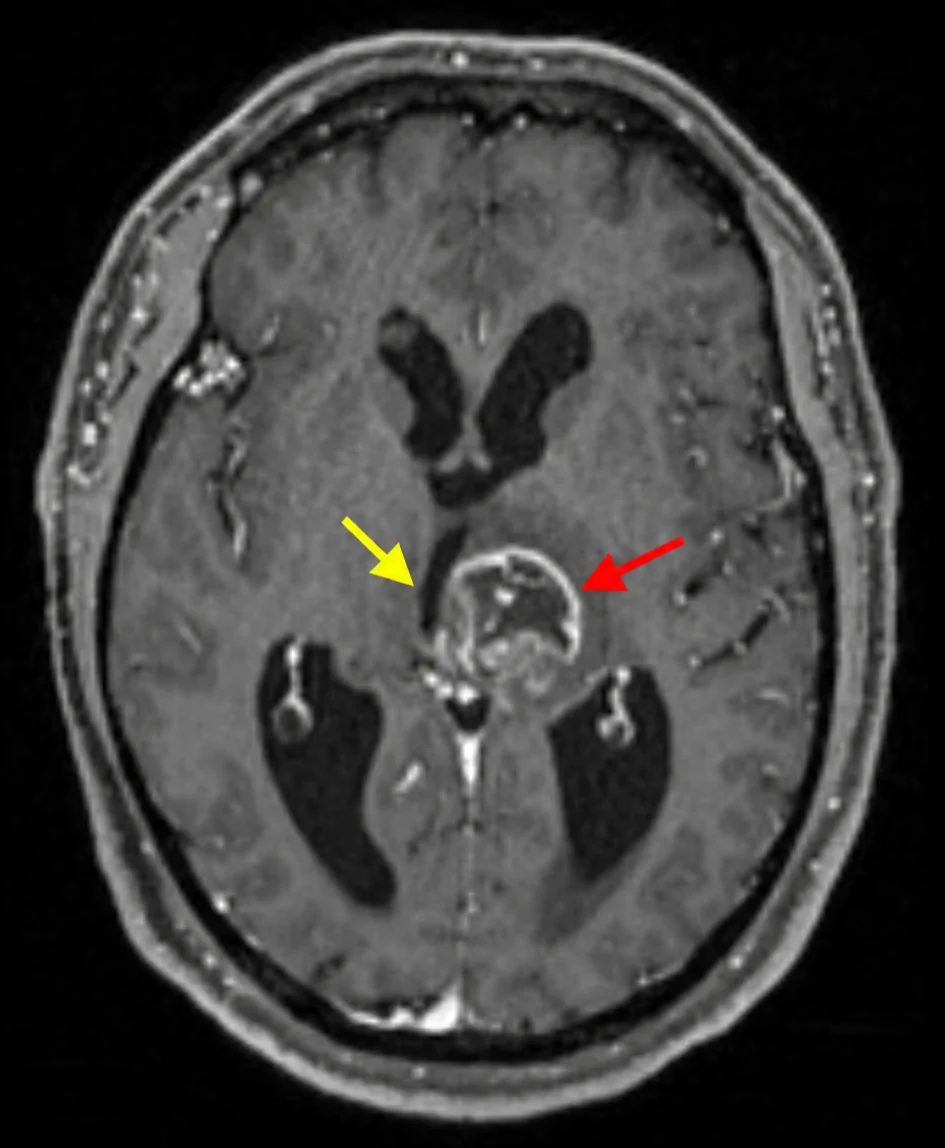
Initial MRI brain, T1-weighted sequence (axial view), demonstrating a predominantly T1-hypointense intra-axial mass centered within the left thalamus with extension into the left midbrain (red arrow). Significant mass effect is present with compression and rightward deviation of the third ventricle (yellow arrow). MRI: magnetic resonance imaging.

**Figure 2:**
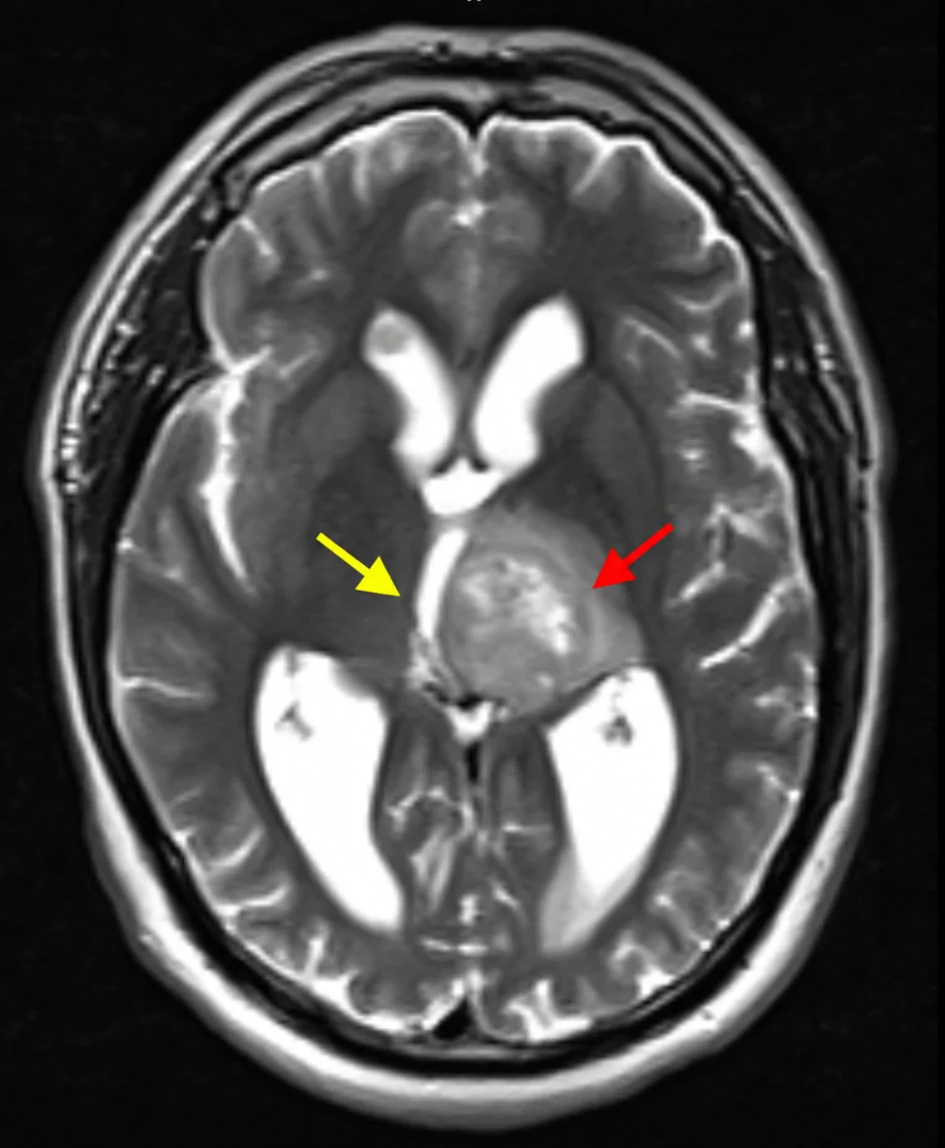
Initial MRI brain, T2-weighted sequence (axial view), demonstrating a heterogeneous left thalamic mass with mixed T2 hypo- and hyperintense signal characteristics (red arrow). Extensive surrounding vasogenic edema is evident, extending into adjacent deep white matter structures (yellow arrow). MRI: magnetic resonance imaging.

**Figure 3:**
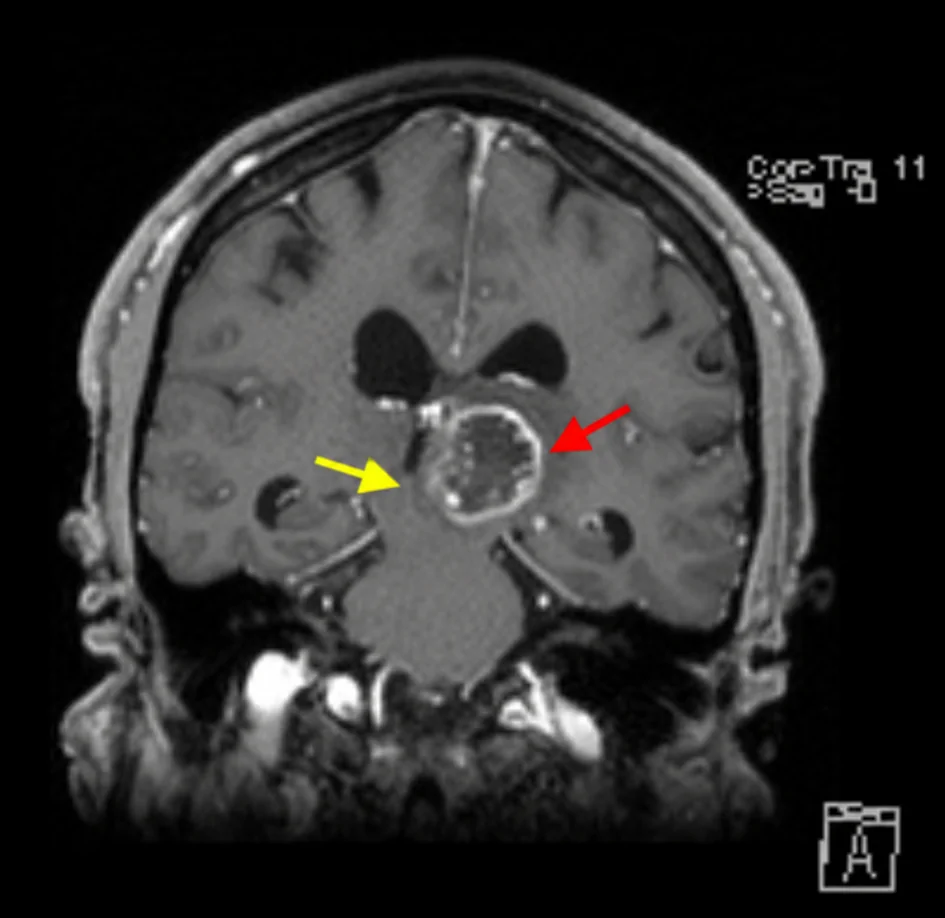
Initial MRI brain, post-contrast T1-weighted sequence (coronal view), showing a heterogeneously enhancing lesion centered within the left thalamus with extension into the midbrain (red arrow). Associated mass effect and third-ventricular compression are demonstrated (yellow arrow). MRI: magnetic resonance imaging.

Given the lesion’s deep-seated location and imaging features concerning for a high-grade glioma, the neurosurgery team recommended an image-guided stereotactic biopsy of the left thalamic mass on day 3 of admission. On day 5, the patient was discharged with close outpatient follow-up with neurosurgery. Histopathologic examination confirmed DMG, H3 K27-altered, WHO grade 4, and an oncology outpatient referral was requested. Tissue quantity was insufficient for O6-methylguanine-DNA-methyltransferase (MGMT) promoter methylation and isocitrate dehydrogenase (IDH)1/IDH2 mutation analysis. Comprehensive genomic profiling performed through Tempus testing identified pathogenic alterations involving *H3F3A* K28M, *KRAS* G12V, and *PHF6*. Variants of uncertain significance were also identified involving *ABCC3*, *EGFR*, and *PDGFRA*. Germline testing did not demonstrate pathogenic variants or variants of uncertain significance.

During the hospitalization, the patient was started on dexamethasone 4 mg every 6 h, which was gradually tapered over the next 5 weeks. Soon after the dexamethasone taper, the patient was evaluated in the medical oncology clinic. The diagnosis, the aggressive nature of the disease, and the rarity of adult DMG were discussed with the patient. Concurrent neurosurgical consultation concluded that operative intervention would carry a substantial risk of significant neurologic deficits due to involvement of the thalamus and midbrain and recommended proceeding with radiation therapy rather than attempted surgical resection.

Radiation oncology recommended definitive external beam radiotherapy to a total dose of 60 Gy, delivered in 30 fractions using volumetric modulated arc therapy (VMAT) with daily image guidance. Radiation therapy was started approximately 10 weeks after the initial presentation and was administered for a total of 6 weeks. Dexamethasone was resumed midway through radiotherapy because the patient experienced worsening lower-extremity weakness and coordination difficulties. Steroids were subsequently tapered successfully and discontinued 3 weeks after radiotherapy concluded. Concurrent temozolomide chemotherapy was not administered, based on available literature demonstrating limited survival benefit of alkylating chemotherapy in DMG, particularly in H3 K27-altered tumors. Radiation monotherapy was favored after multidisciplinary discussion.

Follow-up MRI of the brain, performed 3 weeks (approximately 6 months from diagnosis) after completion of radiotherapy, demonstrated an interval decrease in the size of the heterogeneously enhancing left thalamic lesion extending into the left midbrain, consistent with treatment response (Figs. 4, 5). However, imaging also demonstrated a small subependymal nodule along the floor of the anterior right lateral ventricle near the frontal horn, considered of uncertain significance and concerning for possible early subependymal dissemination (Fig. 6). Repeat MRI 6 weeks after this showed no significant interval change in the left thalamic mass or the right subependymal lesion. Continued surveillance imaging was planned, with repeat MRI scheduled every 3 months.

**Figure 4:**
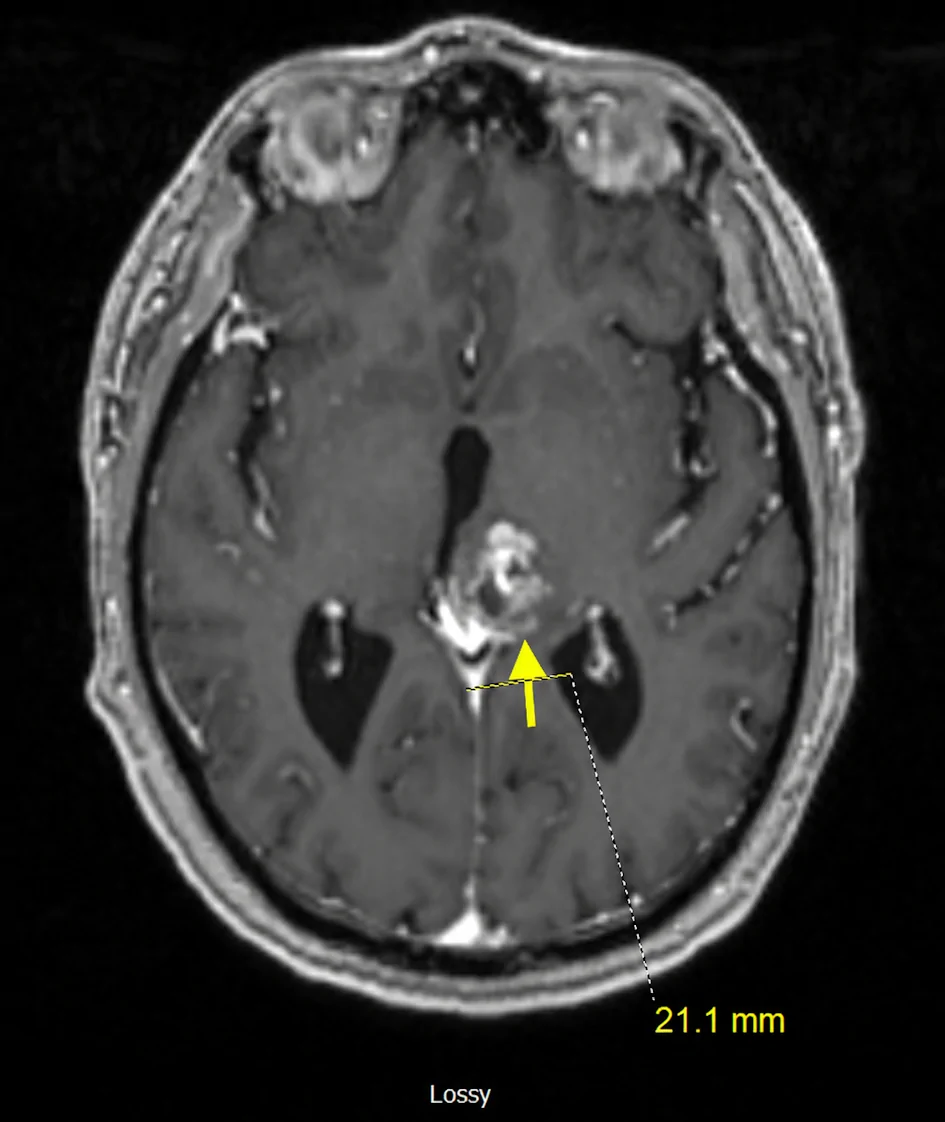
Follow-up MRI brain obtained after completion of radiotherapy, post-contrast T1-weighted sequence (axial view), demonstrating interval reduction in the size of the heterogeneously enhancing left thalamic lesion, consistent with treatment response (yellow arrow). MRI: magnetic resonance imaging.

**Figure 5:**
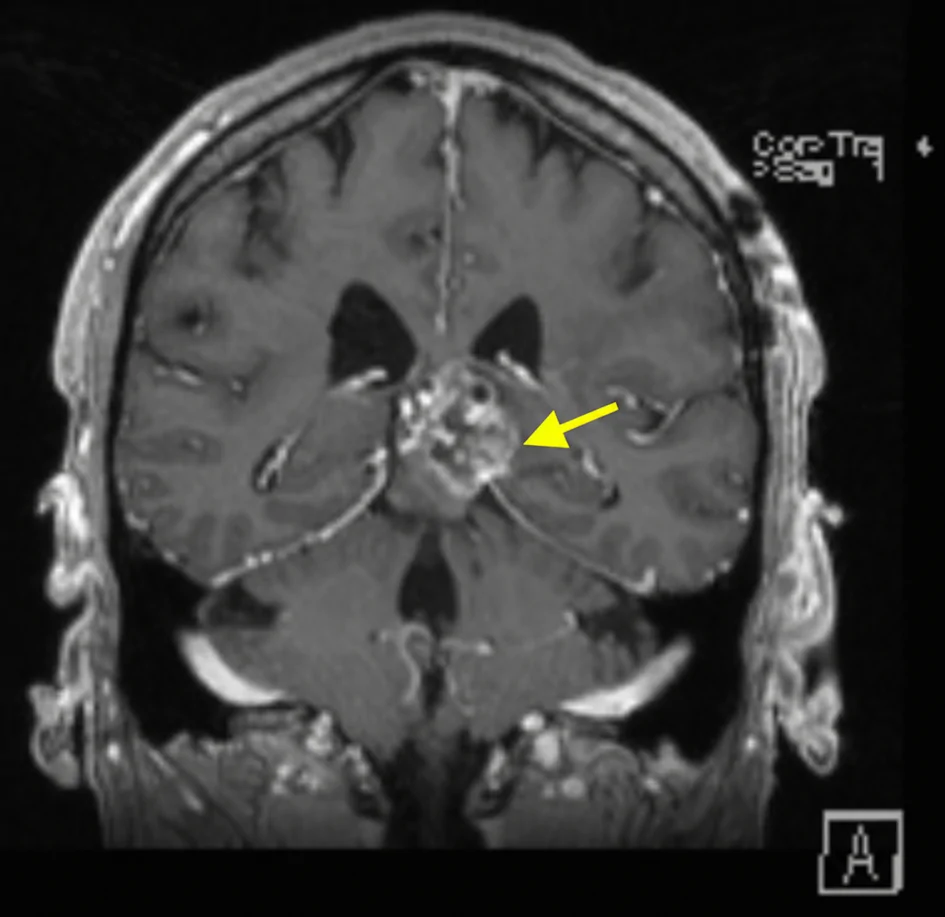
Follow-up MRI brain obtained after completion of radiotherapy, T1-weighted sequence (coronal view), demonstrating interval reduction in the size of the heterogeneously enhancing left thalamic lesion, consistent with treatment response (yellow arrow). MRI: magnetic resonance imaging.

**Figure 6:**
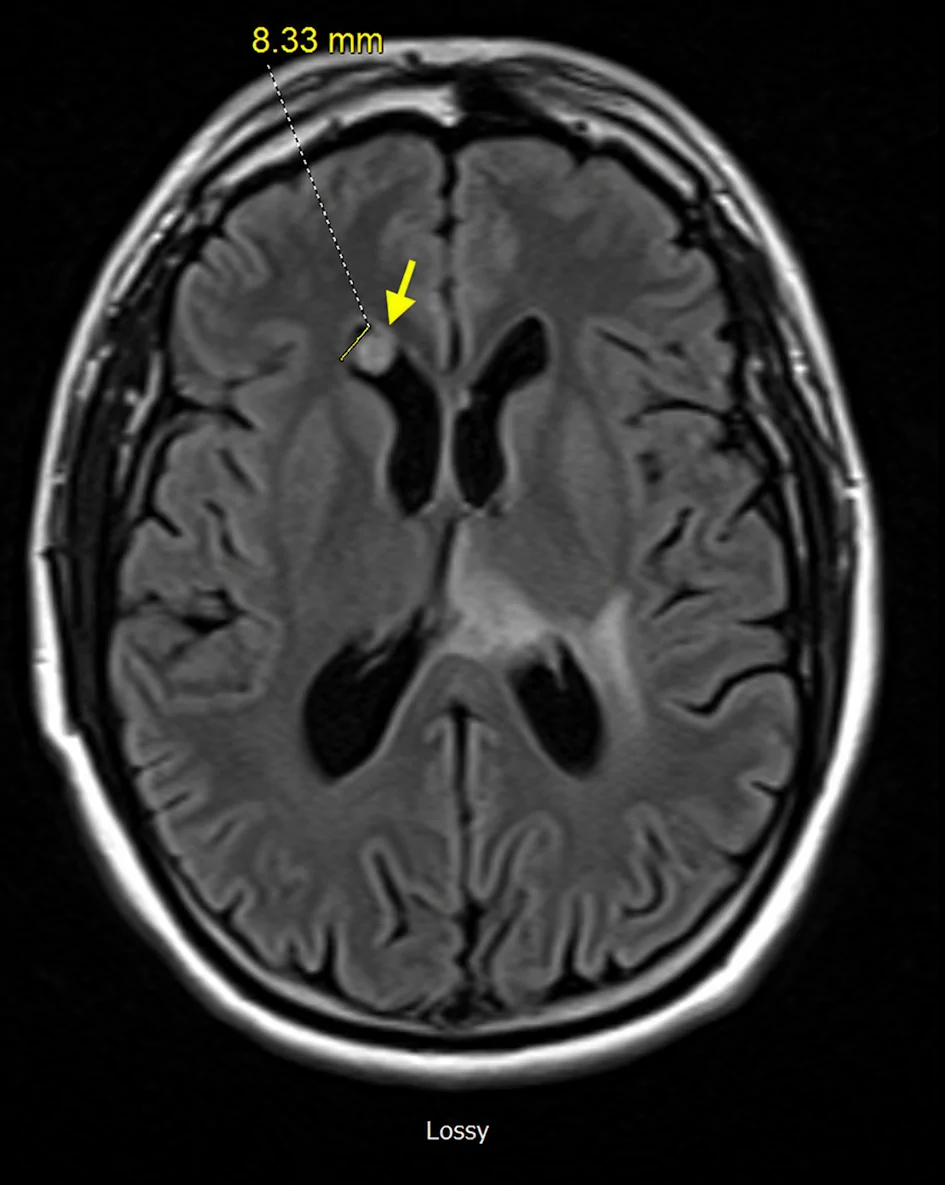
Follow-up MRI brain, post-contrast T1-weighted sequence (axial view), demonstrating a small enhancing subependymal nodule along the floor of the anterior right lateral ventricle near the frontal horn, concerning for possible early subependymal dissemination (yellow arrow). MRI: magnetic resonance imaging.

The patient is currently 8 months from diagnosis and remains asymptomatic and clinically stable. A shared decision was made not to proceed with a subependymal biopsy, and to continue with ongoing surveillance and management plans, given no significant change in the lesion on the recent MRI. The oncology team discussed potential future therapeutic considerations with the patient, based on his clinical progression, including dordaviprone, which received US Food and Drug Administration (FDA) approval in 2025 for H3 K27-altered DMG.

## Discussion

DMG, H3 K27-altered, is one of the rarest and most aggressive primary central nervous system tumors and continues to carry an extremely poor prognosis despite advances in molecular characterization and targeted therapy development [1, 3]. In pediatric cohorts, median survival generally ranges from 9 to 15 months, although adult cases may demonstrate better outcomes [4, 10].

### Adult DMG

Adult DMG remains substantially less common than pediatric disease and remains incompletely characterized [4, 5]. While pediatric DMGs classically arise within the pons, adult tumors more frequently involve the thalamus, spinal cord, and other midline structures [4, 6]. Adult DMGs additionally exhibit greater molecular heterogeneity compared with pediatric disease [5].

Several retrospective studies suggest that adult patients may have slightly better survival than pediatric patients, although outcomes remain poor overall [4, 11]. Meyronet et al evaluated adult H3 K27M-mutant gliomas and found that adult tumors have broader anatomic distribution and distinct molecular characteristics compared with pediatric DIPG [4]. Similarly, Schulte et al demonstrated that adult DMGs show clinicopathologic diversity and variable radiographic features that may complicate diagnosis [5].

The present case demonstrates several features typical of adult DMG, including thalamic location, heterogeneous imaging appearance, H3K27M mutation with additional molecular diversity, and infiltrative extension into adjacent midbrain structures.

### Molecular pathogenesis and histone H3 alterations

Histone H3 alterations involving lysine 27 are the molecular hallmark of DMG [2, 3]. These mutations impair trimethylation of H3K27 by inhibiting polycomb repressive complex 2 (PRC2), resulting in widespread epigenetic dysregulation and oncogenic transcriptional activation [12, 13]. H3 K27 alterations are identified in approximately 15–60% of adult midline gliomas, a considerably lower frequency than the 80–90% reported in pediatric DMG. Emerging evidence suggests that the biological behavior of adult H3 K27-altered DMGs may differ from that of pediatric disease, with some studies reporting longer survival among adults harboring H3 K27M-mutant tumors compared with pediatric cases [6, 7].

The *H3F3A* K28M mutation identified in this patient functionally corresponds to the H3 K27-altered pathway underlying the biology of DMG [2, 12]. Histone H3 mutations are now considered defining diagnostic features regardless of histologic morphology [1]. Solomon et al demonstrated substantial histologic variability among H3 K27-altered gliomas, underscoring the need for molecular profiling for accurate diagnosis [3]. Histopathologic appearances may range from relatively low-grade morphology to highly pleomorphic high-grade astrocytic features [3–5].

### Cytogenetic complexity and diversity of DMG

Current evidence suggests that there are distinct subgroups of DMG harboring various oncogenic driver alterations, with the most common involving receptor tyrosine kinase pathways (*EGFR*, *PDGFRA*, *ACVR1*), DNA repair (*TP53*, *PPM1D*), and chromatin remodeling (*ATRX*) [7–9]. One of the most notable aspects of this case is the identification of concurrent *PHF6* and *KRAS* G12V mutations on molecular profiling, both of which may have biologic and potential therapeutic significance.

The *KRAS* G12V mutation is a well-established activating oncogenic alteration that constitutively stimulates the RAS/MAPK signaling pathway, promoting cellular proliferation, survival, and tumor progression. In pancreatic ductal adenocarcinoma (PDAC), non-small cell lung cancer (NSCLC), and colorectal cancer (CRC), *KRAS* G12V has been associated with aggressive tumor biology and resistance to conventional therapies [10]. Although direct KRAS G12V inhibitors are not yet clinically available, emerging strategies, including MEK inhibition, G12C inhibitors (sotorasib, adagrasib), and KRAS G12V-specific T-cell receptor (TCR)-based immunotherapies, are under active investigation and may offer future treatment opportunities for patients with these molecular alterations [11–13].

While MAPK pathway alterations are increasingly recognized in pediatric low-grade gliomas, their role in DMG remains less clearly defined [9, 14]. Recent studies have identified a subset of H3 K27-altered DMGs harboring MAPK pathway alterations that may exhibit distinct biological behavior and a favorable prognosis and could represent candidates for future targeted therapeutic approaches [9, 11–13].

*PHF6* encodes an X-linked epigenetic transcriptional regulator involved in chromatin remodeling, neurodevelopment, and hematopoiesis. Even though PHF6 alterations (somatic) are best characterized in hematologic malignancies, particularly T-cell acute lymphoblastic leukemia (T-ALL) and mixed phenotype acute leukemia (MPAL), emerging evidence suggests that loss of PHF6 function may contribute to tumor progression in solid tumors through dysregulation of transcriptional and epigenetic pathways [15]. The clinical significance of *PHF6* mutations in DMG remains poorly defined; however, its presence may reflect additional genomic complexity and warrants further investigation.

### Imaging characteristics and differential diagnosis

Radiographically, DMGs often demonstrate infiltrative, T2-hyperintense lesions involving midline structures, with variable enhancement patterns [4, 5, 16]. Adult tumors may exhibit greater radiographic heterogeneity compared with classic pediatric DIPG [5, 16]. The lesion in this patient demonstrated heterogeneous enhancement, surrounding edema, and significant mass effect upon the third ventricle.

Differential considerations for adult thalamic masses include glioblastoma, primary central nervous system (CNS) lymphoma, metastasis, demyelinating disease, germ cell tumor, and inflammatory lesions [5, 16]. Recognition of DMG in adult patients is important because diagnosis increasingly depends upon molecular profiling rather than imaging or histology alone [2–5, 16].

### Therapeutic considerations

Management of DMG remains challenging. Surgical resection is typically infeasible in most cases because of infiltrative growth within eloquent midline structures. Management of DMG focuses largely on symptom control and maintaining quality of life. Corticosteroids, particularly dexamethasone, are commonly used to reduce peritumoral edema and rapidly improve neurological symptoms, although they do not affect tumor progression [17]. Additional supportive measures may include bevacizumab [18] for steroid-refractory edema or radiation necrosis, and cerebrospinal fluid diversion procedures, such as ventriculoperitoneal shunting or endoscopic third ventriculostomy, for patients who develop obstructive hydrocephalus [19–21].

Radiation therapy remains the cornerstone of treatment and is the only modality proven to temporarily alter the natural history of DMG. Standard external-beam radiotherapy, typically delivered to a total dose of 54–59.4 Gy, yields transient radiographic responses and symptomatic improvement in many patients [22–24]. Hypofractionated regimens offer similar outcomes with shorter treatment durations and may reduce treatment burden, particularly in younger children [23]. Despite decades of investigation, strategies such as radiation dose escalation, altered fractionation schedules, radiosensitizers, and concurrent chemotherapy have failed to demonstrate superiority over conventional radiation therapy [25–28]. Nevertheless, radiation remains the standard first-line treatment because of its consistent palliative benefit [22, 25–28].

For recurrent or progressive disease, therapeutic options remain limited. Reirradiation may offer temporary symptomatic relief and modest survival benefit in selected patients [29, 30]. In 2025, dordaviprone (ONC201) became the first FDA-approved systemic therapy for recurrent or progressive H3 K27M-mutant DMG, although data on pontine tumors remain limited [31, 32]. Approval of dordaviprone has significantly altered the therapeutic landscape for adult DMG, and early clinical studies have demonstrated durable responses and prolonged survival in select patients [31, 32]. Conventional chemotherapy, including temozolomide-based regimens, has not demonstrated meaningful survival benefit and is not routinely recommended outside clinical trials [33–36]. Given the lack of convincing evidence of chemotherapy benefit and concerns about treatment-related toxicity, omitting temozolomide in the present case was considered reasonable.

Current research efforts are focused on molecularly targeted and immunotherapeutic approaches, including H3 K27M peptide vaccines [37], disialoganglioside 2 (GD2)- and B7 homolog 3 protein (B7-H3)-directed chimeric antigen receptor T-cell (CAR-T) therapies [38–41], oncolytic viruses [42], and convection-enhanced delivery of radioimmunotheranostics [43], which have shown encouraging early results but remain investigational. Potential therapies targeting KRAS/MPAK pathways are also briefly discussed in the molecular section above [11–13].

### Possible early subependymal dissemination

An additional important feature in this case is the presence of a small subependymal ventricular nodule identified on follow-up MRI. Dissemination through cerebrospinal fluid pathways has increasingly been recognized in DMG [35, 44]. Leptomeningeal and ventricular dissemination may occur at diagnosis or during disease progression and generally portends a poor prognosis [35, 44, 45]. Panditharatna et al demonstrated that DMG frequently shows evidence of CSF tumor dissemination detectable by liquid biopsy [44]. Although the significance of the ventricular nodule in this case remains uncertain, close surveillance is warranted given the possibility of early subependymal spread.

### Prognosis

H3 K27-altered DMGs remain among the most aggressive pediatric central nervous system tumors despite advances in molecular characterization and management, with a mean survival of approximately 8–10 months, a median survival of approximately 9–15 months, and a 5-year survival rate below 3%. However, the prognosis in adult DMG is variable and generally favorable, with mean survival reported to range from 10 to 30 months across various studies [2–5]. Long-term survivors frequently experience significant neurocognitive sequelae related to both the disease and its treatment [5, 14, 45].

Several clinical factors have been associated with relatively improved outcomes, including age at diagnosis < 3 years or > 10 years in the pediatric population and age < 60 years in adults; longer duration of symptoms before diagnosis; high performance status; absence of cranial nerve deficits; atypical imaging features (minimal or absent contrast enhancement); and the possibility and extent of resection (total resection) [2, 5, 14, 16, 35, 45, 46].

Beyond clinical factors, increasing evidence indicates that specific molecular alterations may confer a survival advantage in patients with H3 K27-altered DMGs. The most consistently reported favorable concurrent biomarkers include alterations in *ACVR1*, the MAPK pathway (*FGFR1*, *BRAF* (V600E), *KRAS*), and H3.1 K27M. *PDGFRA*, *TP53*, and *EGFR* alterations generally do not confer a survival advantage and are often associated with more aggressive disease [2, 14, 16, 19, 45].

### Conclusions

Adult thalamic DMG, H3 K27-altered, remains a rare and highly aggressive CNS malignancy with limited therapeutic options. This case highlights several clinically important features, including adult presentation, deep thalamic involvement with brainstem extension, radiographic heterogeneity, comutations of *KRAS* G12V and *PHF6*, variations in *PDFGRA* and possible early subependymal dissemination. Comprehensive molecular characterization continues to refine understanding of DMG biology and may ultimately facilitate the development of targeted therapeutic strategies. Further investigation into the prognostic and therapeutic implications of PHF6, KRAS and PDGFRA pathway alterations in DMG is warranted.

### Learning points

Adult DMG, H3 K27-altered, is a rare WHO grade 4 CNS tumor that differs from pediatric DMG in its anatomical distribution and molecular heterogeneity. Comprehensive molecular profiling is essential, as uncommon co-occurring alterations, such as *KRAS* G12V and *PHF6*, may provide insights into tumor biology and future therapeutic opportunities. Although radiation therapy remains the cornerstone of treatment, emerging targeted therapies and immunotherapeutic approaches are reshaping the management landscape. The presence of a possible subependymal lesion in this case further highlights the importance of long-term surveillance for ventricular and cerebrospinal fluid dissemination.

## Data Availability

The authors declare that the data supporting the findings of this case report are available within the article. All data analyzed during this study were obtained from previously published studies and are available in the public domain.

## Abbreviations

DMG: diffuse midline glioma
DIPG: diffuse intrinsic pontine glioma
WHO: World Health Organization
CNS: central nervous system
MRI: magnetic resonance imaging
CT: computed tomography
VMAT: volumetric modulated arc therapy
CSF: cerebrospinal fluid
GERD: gastroesophageal reflux disease
H3F3A: H3 histone family member 3A
KRAS: Kirsten rat sarcoma viral oncogene homolog
PHF6: plant homeodomain finger protein 6
EGFR: epidermal growth factor receptor
PDGFRA: platelet-derived growth factor receptor alpha
ATRX: alpha-thalassemia/mental retardation syndrome X-linked
TP53: tumor protein p53
ACVR1: activin A receptor type 1
PPM1D: protein phosphatase, Mg^2+^/Mn^2+^-dependent 1D
MAPK: mitogen-activated protein kinase
PRC2: polycomb repressive complex 2
PDAC: pancreatic ductal adenocarcinoma
NSCLC: non-small cell lung cancer
CRC: colorectal cancer
TCR: T-cell receptor
T-ALL: T-cell acute lymphoblastic leukemia
MPAL: mixed phenotype acute leukemia
FDA: Food and Drug Administration
GD2: disialoganglioside 2
B7-H3: B7 homolog 3 protein
